# Interleukin 7 from Maternal Milk Crosses the Intestinal Barrier and Modulates T-Cell Development in Offspring

**DOI:** 10.1371/journal.pone.0020812

**Published:** 2011-06-30

**Authors:** Richard Aspinall, Andrew M. Prentice, Pa Tamba Ngom

**Affiliations:** 1 Department of Immunology, Imperial College London, London, United Kingdom; 2 Nutrition Programme, Medical Research Council Laboratories, Serrekunda, The Gambia; 3 Medical Research Council International Nutrition Group, London School of Hygiene and Tropical Medicine, London, United Kingdom; 4 Cranfield Health, Cranfield University, Bedfordshire, United Kingdom; French National Centre for Scientific Research, France

## Abstract

**Background:**

Breastfeeding protects against illnesses and death in hazardous environments, an effect partly mediated by improved immune function. One hypothesis suggests that factors within milk supplement the inadequate immune response of the offspring, but this has not been able to account for a series of observations showing that factors within maternally derived milk may supplement the development of the immune system through a direct effect on the primary lymphoid organs. In a previous human study we reported evidence suggesting a link between IL-7 in breast milk and the thymic output of infants. Here we report evidence in mice of direct action of maternally-derived IL-7 on T cell development in the offspring.

**Methods and Findings:**

We have used recombinant IL-7 labelled with a fluorescent dye to trace the movement in live mice of IL-7 from the stomach across the gut and into the lymphoid tissues. To validate the functional ability of maternally derived IL-7 we cross fostered IL-7 knock-out mice onto normal wild type mothers. Subsets of thymocytes and populations of peripheral T cells were significantly higher than those found in knock-out mice receiving milk from IL-7 knock-out mothers.

**Conclusions/Significance:**

Our study provides direct evidence that interleukin 7, a factor which is critical in the development of T lymphocytes, when maternally derived can transfer across the intestine of the offspring, increase T cell production in the thymus and support the survival of T cells in the peripheral secondary lymphoid tissue.

## Introduction

Breastfeeding, according to the World Health Organisation is an unequalled way of providing ideal food for the healthy growth and development of infants; who should be exclusively breastfed for the first six months of life [1]. Epidemiologic studies in low income countries show that breast feeding substantially reduces the risk of infection, especially from enteric disease [2], [3] and that lactation represents an ‘ingenious immunological integration of the mother and the child’ [2]. Breast milk is also a vehicle for providing immunological support to the developing offspring with the well demonstrated presence of the products of both cellular and humoral immune responses in breast milk following vaccination of the mother [4], [5], [6], [7], [8], [9], [10], [11].

The reduction in the severity and frequency of disease in breast-fed infants indicates the impact of components of breast milk on immunity in the offspring, but at issue is whether this protection arises solely through factors in the milk supplementing the inadequate immune response of the offspring or whether components within milk have a dichotomic effect both supplementing the response and enhancing the development of the immune system, boosting the production and aiding the survival of lymphocytes, in the offspring.

Supplementation of the immune response of the offspring is the current paradigm for characterising the beneficial action of breastfeeding with evidence presented for factors transmitted to the offspring acting directly on the potential pathogen. For example IgA antibodies specific for *E. coli, H. influenzae, H. pylori* and *S. pneumoniae* which have been found in human milk and which act in the intestinal lumen (or in the airways following inhalation of milk droplets) of the offspring by binding antigen, so reducing the infective nature of potential pathogens. In addition factors such as epidermal growth factor may help to induce more rapid maturation of the intestinal epithelium leading to decreased permeability to pathogens [12].

Less direct evidence is available to support the notion that breast milk factors enhance the development of the immune system within the offspring. Whilst the link between inadequate nutrition and thymic atrophy has been known since at least 1810 [13] it was only more recently shown that offspring fed on breast milk possessed larger thymuses than those fed on formula feed [14], [15] suggesting the presence of a component in breast milk which could augment thymic growth and development. Thymic size is important, partly because in humans a small thymic size at 6 months of age is associated with a higher risk of mortality [16] and partly because thymic size is also an indicator of thymic output [17]. A positive correlation between thymic size in the perinatal period and survival was also shown by a study in Guinea-Bissau [18] and later extended by work in rural Gambia [19].

Studies in rural Gambia showed that children born in the hungry season have smaller thymuses eight weeks after birth compared with their counterparts born in the harvest season [19], [20]. Moreover the rates of mortality in young adulthood of those born before the harvest were up to 10-fold higher than their counterparts, with the available data suggesting many of the deaths were infection-related [21], [22]. One factor considered integral to the concept of enhancing immune development was interleukin-7 (IL-7) shown to be present at higher levels in the breast milk of those born after the harvest in the rural Gambian study [19]. IL-7 has a central role in T cell development in the thymus and the maintenance of T cells in the peripheral T cell pool [23]. Because of the association between the amount of available IL-7 in the thymus and thymocyte numbers [24], the possibility that maternally-derived IL-7 was having an additive effect on thymic output in the offspring has been proposed [19]. However this would require that the IL-7 survive digestion and be transmitted from the mother to the tissues of the offspring through the medium of the maternally derived milk.

We sought to test this possibility by feeding newborn mouse pups recombinant IL-7, labelled with a fluorescent dye, and tracing its path within the animal. To supplement these studies we also sought to determine the effect of cross fostering newborn transgenic mouse pups carrying the IL-7 knock out gene onto wild type dams to determine whether there was an effect of the murine maternally derived IL-7.

## Methods

### Ethics statement

All mice were housed in the animal house at Imperial College London and all experiments were carried out under approval by the Home Office under Project License (PPL70/5736). All efforts were made to minimise pain and suffering. No human samples were used in this study.

### Mice

Wild type C57Bl/6 mice were purchased from Harlan Olac and maintained either in positive pressure isolators or individually ventilated cages. For feeding experiments most mice were born at night and so the day of their first appearance was considered to be day 1. IL-7 knock-out mice were obtained from the National Institute of Medical Research with the permission of Prof. Paulo Viera and maintained in positive pressure isolators. These mice were originally generated from mutated ES cells injected into blastocysts from C57BL/6 mice, where the resulting male chimeras were mated to C57BL/6 females to test for germ-line transmission [25].

### Analysis of IL-7 levels in milk

Sucking pups were culled by cervical dislocation, their stomachs removed and the wall dissected away from the bolus of milk. The partially solidified milk was resuspended in phosphate buffered saline overnight at 4°C and the IL-7 content was assayed by sandwich ELISA and quantified against recombinant murine IL-7 standards (R&D, UK).

### Cell staining and analysis

Analysis of thymocyte subsets was performed as previously described [26]. Briefly the thymus was removed and cell suspensions made and approximately 1×10^6^ thymocytes were incubated with the following sets of anti-bodies; anti-CD4-R-PE (clone H129.19), anti-CD8-R-PE (clone 53-6.7), anti-CD3-R-PE (clone 17A2), anti-CD19-R-PE (clone ID3), anti-CD25-FITC (clone 7D4) and anti-CD44-Cy-chrome (clone IM7) or their appropriate isotype controls. Alternatively thymocytes were stained with anti-CD8-FITC (clone 53-6.7) and anti-CD4-Cy-Chrome (clone H129.19) or their appropriate isotype controls. Spleens were removed and cell suspensions made and the total number of cells obtained counted. Cells were then stained with anti-CD8-FITC (clone 53-6.7) and anti-CD4-Cy-Chrome (clone H129.19) and anti-CD3-R-PE (clone 17A2) or their appropriate isotype controls. All antibodies were obtained from Becton Dickinson.

Cells were stained with the antibody cocktails for approximately 30 minutes at 4°C and then washed with phosphate buffered saline (PBS) and fixed with 1% paraformaldehyde in PBS. Cells were analysed with a with a FACS Calibur flow cytometer (BD Pharmingen) and data analysis was performed using WinMDI software.

### IRDye 800 labelling of IL-7 and tracing in vivo

Carrier-free recombinant mouse IL-7 (R&D) or carrier-free recombinant mouse albumin (Gentaur, Brussels) were labelled with IRDye800 (Li-cor biosciences) according to the manufacturer's instructions a version of which can be found at http://biosupport.licor.com/support. Briefly, the protein dissolved in PBS and the reactive IRDye 800 dissolved in DMSO were mixed in the dark for approximately 2 hours and then dialysed against PBS. 100 ng of labelled proteins were delivered into the stomachs of newborn mice within 24 hours of birth by gavage and the mice were immobilised by chilling [27] and scanned using a Li-cor Odyssey scanner.

### Quantitation of labelled protein in the tissues

Tissues were digested in 50 µl of a 1 in 5 dilution of proteinase k (Qiagen UK) in PBS overnight at 37°C. The solution was then placed in the wells of a 96 well plate and scanned. The plate also contained a dilution series in quadruplicate of labelled mouse IL-7. Prior analysis revealed that overnight incubation in the presence of proteinase K did not affect the level of fluorescence.

### Cross fostering experiments

Breeding programs were set up with both the IL-7 knock out and C57/Bl6 wild type animals. Litters were transferred to their foster dams within 24 hours of birth. Animals were taken at weaning (approximately 20 days after birth) culled by CO_2_ asphyxiation and their tissues removed.

### Statistical analysis

Statistical analysis was performed using an unpaired T test where significance was considered when p<0.05.

## Results

### IL-7 in mouse milk

**Figure 1:**
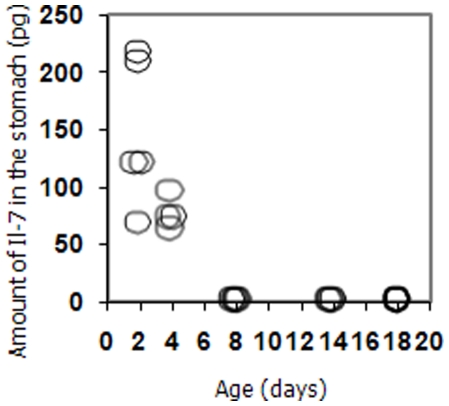
IL-7 can be detected in the milk of mice in the offspring after birth but not for the entire period of weaning. The IL-7 content of milk was assayed by sandwich ELISA and quantified against recombinant murine IL-7 standards.

We demonstrated the presence of IL-7 in mouse milk, by assaying milk obtained from the stomachs of suckling mice at several time points in the post natal period. Two days after birth, mouse pups had IL-7 present at an average of 146 pg per animal and this fell to an average of 76pg per animal 4 days after birth and was below the level of detection 8 days after birth (Fig 1). We tested animals at both 14 and 18 days after birth but failed to detect IL-7 in the milk in their stomachs at these times. We validated the specificity of our assay by testing milk from IL-7 knock-out mice and found it to be negative and were further concerned that the lL-7 we detected in milk was absorbed from the plasma. This seemed unlikely because we could detect IL-7 in the blood of these animals at weaning at an average of 13.6 ng/ml of serum but were unable to detect any in the stomach at day 8 and thereafter.

### Tracing IL-7 movement from the gut

**Figure 2:**
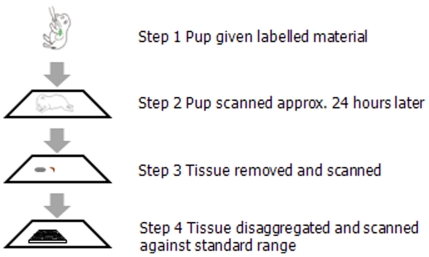
Diagrammatic representation of an outline of the experimental procedure to follow labelled IL-7 from the gut to the lymphoid organs.

**Figure 3:**
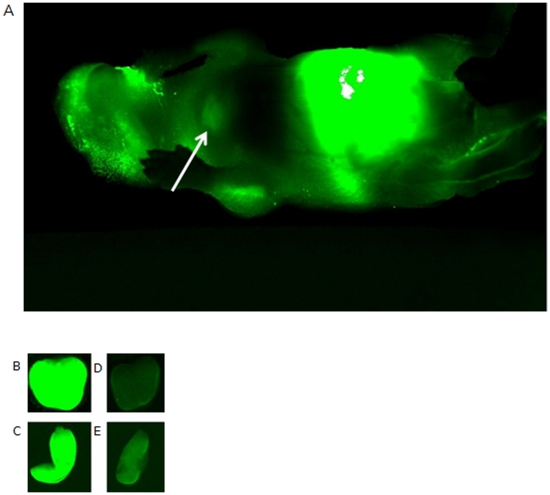
IRDye800 labelled IL-7 introduced by gavage into the stomach of a mouse pup can be detected as green fluorescence in the anterior mediastinum on the next day (A), as indicated by the white arrow. Labelled product is apparent also around the jaws of the animal and in the intestine. The pup is in a prone position on the glass surface of the Odyssey and the image is from underneath. Two examples of samples from quantitation studies are shown here where newborn pups were fed 100 ng of labelled protein, either rIL-7 (B,C) or albumin (D,E), and culled after 24 hours and their thymus (B,D) and spleen (C,E) removed and scanned.

Whether the movement of IL-7 across the gut into the tissues was feasible was tested by introducing recombinant murine IL-7 labelled with IRDye800 into the stomach of newborn mice and tracing its movement as outlined (Fig 2). We following labelled IL-7 in live mouse pups over time by imaging and our initial results suggested that labelled IL-7 could traverse the gut and be identified in the anterior mediastinum in a region where we would expect to find the thymus (Fig 3A).

**Figure 4:**
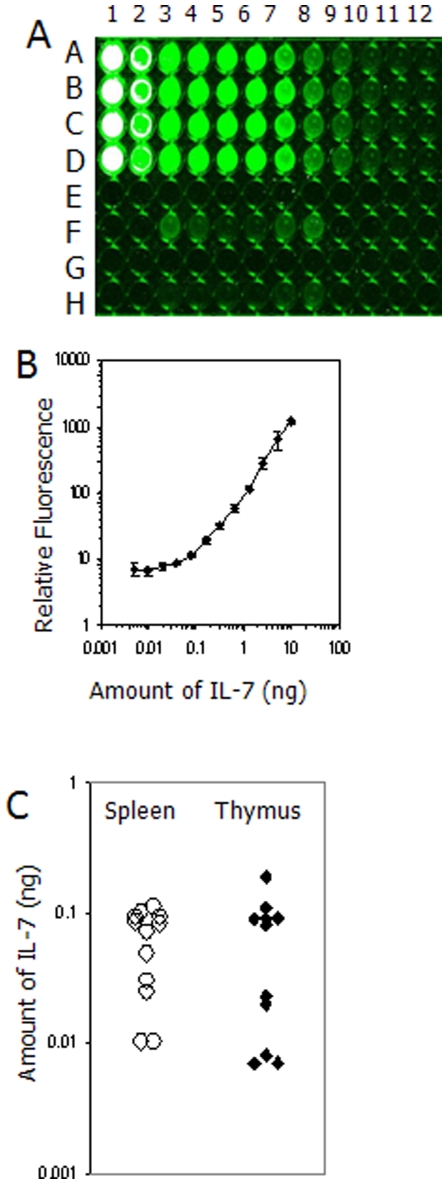
Scan of 96 well plate containing digested tissues from animals given labelled IL-7 and a serial dilution in quadruplicate of labelled murine IL-7 seen in (A). Digested spleens are in row F, wells 3 through 8, and digested thymus tissue is in row H, wells 3 through 8. Wells A–D in column 1 contains IL-7 at 10 ng in a total volume of 50 µl and the subsequent columns (2 through 12) contain doubling serial dilutions. The standard curve of recombinant CW800 labelled murine IL-7 (B) shows means± 1 standard deviation from quadruplicate wells and the calculated amount of IL-7 in individual spleens and thymuses, are seen in (C).

We were concerned that the fluorescence we were detecting in the anterior mediastinum may have derived from label detaching from the IL-7 and to test for this we fed mice either recombinant mouse albumin labelled with IRDye 800 or recombinant mouse IL-7 labelled with the same dye and imaged them over the same time period. When we viewed their fluorescence our results revealed that organs from animals fed labelled IL-7 were far brighter than those fed labelled albumin (Fig 3B-E) suggesting that the IRDye800 remained associated with the IL-7 after its passage through the gut. To assess the percentage of IL-7 which travelled from the gut to the lymphoid organs we fed 1 day old pups 100 ng of labelled IL-7 and 24 hours later excised the thymus and the spleens of these animals. To assess the amount of labelled protein present in these tissues we digested the spleen and thymus from the offspring with proteinase K and measured the fluorescence of the solution, and compared it with our labelled IL-7 standard curve (Fig 4a, b). From this we calculated that the thymus contained on average 65pg (n = 11) whilst the spleen contained an average of 62pg (n = 12) of labelled IL-7 (Fig 4c).

### Cross fostering experiments

Transmission across the gut does not necessarily imply that the maternally-derived IL-7 would be functionally active in the lymphoid tissue, so we sought to determine the contribution of IL-7 in murine milk to T cell production and maintenance in the offspring by analysing IL-7 knock out pups fostered onto wild type dams (KO→WT) and comparing them with IL-7 knock outs on IL-7 knock out dams (KO→KO), wild type on IL-7 knock outs dams (WT→KO) and wild type on wild type dams (WT→WT). Analysis of the subpopulations within the thymus (Table 1) showed similar numbers of triple negative cells in KO→WT, WT→KO and WT→WT pups and these values were almost 3 times higher than found in KO→KO pups. Comparison of later stages in the pathway showed a similar number of double positive and single positive thymocytes in the KO→KO and KO→WT pups and that these values were considerably less than in the WT→KO and WT→WT pups.

**Table 1:** Comparison of the numbers of cells in the major populations of the thymus in offspring and the effect of IL-7.

Condition	n	Number of TN thymocytes (average ± 1SD)×10^6^	Number of CD4^+^CD8^+^thymocytes (average ± 1SD)×10^7^	Total number of CD4^+^CD8^−^ thymocytes (average ± 1SD)×10^7^	Total number of CD4^−^CD8^+^ thymocytes (average ± 1SD)×10^6^
KO to KO	18	0.83±0.87	1.73±1.48	0.38±0.49	1.23±1.8
KO to WT	18	2.36±4.52	1.14±0.49	0.71±0.90	1.5±1.99
WT to KO	10	3.3±4.57	12.7±7.6	3.77±1.73	11.13±5.06
WT to WT	13	2.62±1.33	8.5±6.7	1.4±1.0	3.83±2.84

**Figure 5:**
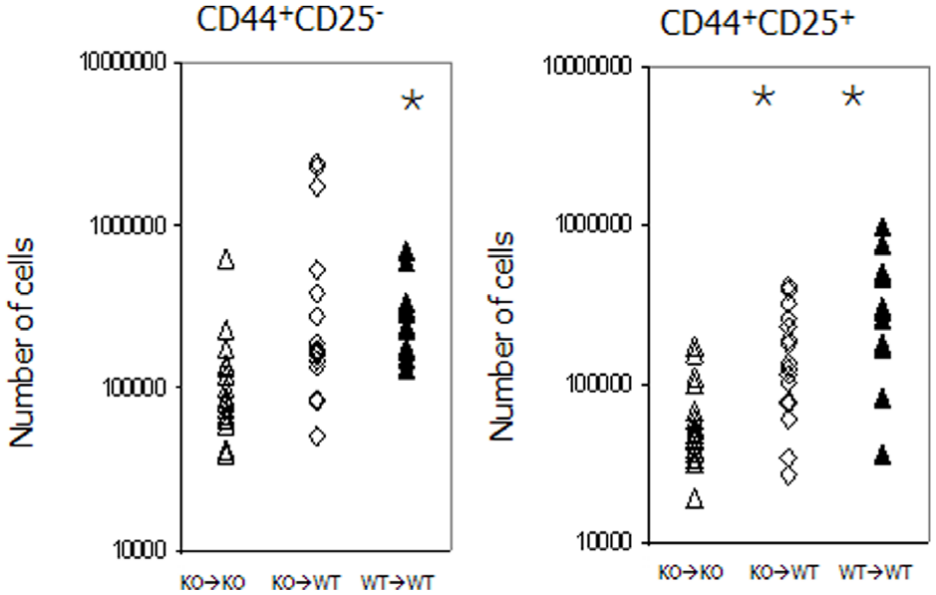
In cross fostering experiments more CD44^+^CD25^−^ and CD44^+^CD25^+^ thymocytes are found in the thymus of IL-7 knock out animals fostered onto wild type animals (⋄) than those left with their IL-7 knock out mothers (▵). Values are similar to those obtained with wild type animals maintained on wild type mothers (▴). Values which are significantly different (where p<0.05) from the knock out animals on knock out mothers are indicated by the 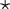
annotation.

Further analysis was undertaken within the triple negative population to determine any differences and the results revealed that there were more CD44^+^CD25^−^ cells in the KO→WT pups (average of 5.05×10^5^) compared with the KO→KO pups (average of 1.5×10^5^), and the progeny of this population, the CD44^+^CD25^+^ cells showed significantly greater numbers in KO→WT animals compared with the KO→KO pups (1.15×10^5^ versus 8.13×10^4^ p = 0.03 Fig 5).

**Figure 6:**
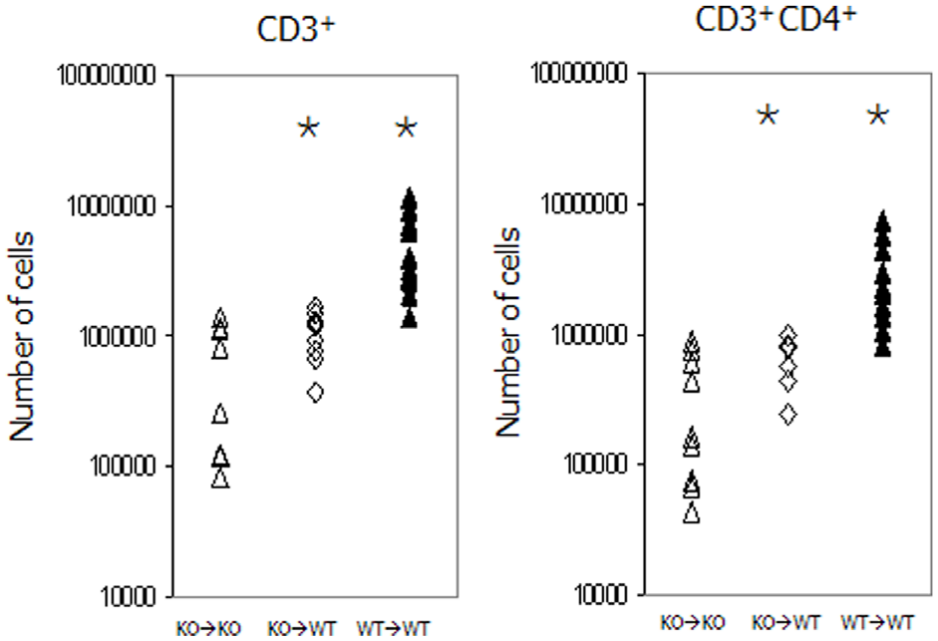
In cross fostering experiments IL-7 knock out animals fostered onto wild type animals (⋄) had more CD3^+^ T cells and more CD3^+^CD4^+^ T cells than those left with their IL-7 knock out mothers (▵). Values are similar to those obtained with wild type animals maintained on wild type mothers (▴).Values which are significantly different (where p<0.05) from the knock out animals on knock out mothers are indicated by the 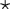
annotation.

To determine the effect of maternal IL-7 on peripheral T cell populations we followed changes in the T cells in the spleens of our KO→WT and compared them with their KO→KO and wild type (WT) counterparts. The results reveal a significantly greater percentage of CD3^+^ T cells in the spleens of KO→WT than in the KO→KO pups and this translated into significantly greater T cell numbers (Table 2). Closer analysis of the subpopulations revealed (Fig 6) that the KO→WT pups contained significantly more CD3^+^CD4^+^ T cells than the KO→KO spleens (6.8×10^5^ vs 3.5×10^5^ p = 0.026).

**Table 2:** Comparison of the numbers of cells in the spleen of offspring and the effect of IL-7.

Condition	Percentage and No. (average ± 1SD×10^6^) of CD3^+^ cells in the spleen [n]
KO to KO	2% (0.59±0.52) [9]
KO to WT	4% (1.09±0.39) [10]
WT to KO	12% (11.8±6.69) [5]
WT to WT	15% (4.86±3.26) [11]

## Discussion

These results demonstrate that IL-7 is present in milk and provide direct evidence that when transmitted to the offspring IL-7 can cross the gut and enter the tissues and impact on both T cell production and T cell maintenance.

The detection of IL-7 in mouse milk mirrors previous human studies [28], [19] and the detection profile of IL-7 within the milk, high after birth and then declining with time is similar to that seen in our earlier human studies in The Gambia [19], and to that reported for IL-18 [29]. The identification of IL-7 in maternally derived milk posed the question of whether it could be transported into the tissues to act systemically. Maturation of the gastrointestinal tract is a dynamic process and at birth the barrier function is still developing. The permeability of the gut to macromolecules is a recognised phenomenon with evidence from previous studies showing that intact large peptides present in milk such as insulin or epidermal growth factor can cross the intestinal barrier and enter the circulation of the offspring [30], [31].

Our initial profiles of the fluorescence distribution after feeding tagged IL-7 suggest that the IL-7 could pass through the gut and enter the tissues however these profiles could also be interpreted as being due to the dissociation of the fluorescent tag from the IL-7 molecule following acid or protease digestion. Secretion of acid is initiated 4 days after birth in the mouse [32] whilst enteropeptidases such as enterokinase are present on postnatal days 2–6 albeit at low levels [33]. The neonates were fed labelled IL-7 on the day after birth and so it seemed unlikely that digestion or dissociation of the protein could be construed to account for the fluorescence seen in the tissues. Moreover the considerably reduced fluorescence seen when animals were fed labelled recombinant murine albumin would also seem to support this and discount the notion that the fluorescent profile we saw was due to digestion of the protein and release of the fluorescent tag.

The data from fluorescent analysis provides direct support for the movement of IL-7 into the tissues, and further confirmation that IL-7 could cross the gut and be functionally active in the tissues came from analysis of lymphoid tissue in IL-7 knock out pups fostered onto wild type dams at birth.

Lymphocyte development and maintenance is severely compromised in IL-7 knockout mice which display severe lymphopoenia with the spleen and thymus having less than 20% of the cellularity of normal wild type controls [25]. In the thymus of normal wild type animals the earliest precursors of the T cell pathway are CD3^−^CD4l
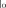
CD8 [34] and differentiation stages from these precursors can be discriminated on the basis of expression of CD44 and CD25. Progress from the most immature stage, CD44^+^CD25^−^ requires the transient acquisition of CD25 so the cell first becomes CD44^+^CD25^+^ before becoming CD44^−^CD25^+^ and then the loss of CD44 when the population is CD44^−^CD25^−^ [35]. IL-7 has a crucial role in these early developmental stages where it aids the survival of cells [36] and all subsets express the IL-7 receptor but at different levels. Because early stages in the intrathymic pathway show slightly higher levels of expression of the IL-7 receptor than the later CD44^−^ stages [37] their enhanced survival may be favoured when IL-7 is limited and this is reflected in the differences seen between the KO pups on KO dams and those on WT dams. The increase in the number of cells in the populations of these early stages of the T cell pathway seen in the KO pups on WT dams compared with KO pups on KO dams was noticeable and we considered sample bias to be an unlikely contributor to this because for each condition we analysed the results of several transfers.

Analysis of the T cells in the spleen was undertaken to confirm whether there were any downstream effects on T cell development. The significant increase in the number of T cells within the CD4^+^ subset in the KO pups on WT dams compared with KO pups on KO dams fits with previous work showing that the naive CD4^+^ T cell population is the compartment easily perturbed by abrogating IL-7–IL-7R interactions, which may be because this population expresses a slightly higher level of IL-7R on the cell surface than other peripheral T cell populations [38]. Our results also fit with a previous study which noted that during the early postnatal period recent thymic emigrants emerge into a lymphopenic environment and undergo antigen independent proliferation which is mainly restricted to CD4^+^ T cells [39].

The results from both the tracing experiments and the cross fostering experiments together provide direct evidence that maternally derived IL-7 impacts on the immune system in the offspring. Breast feeding has been known to have a positive effect on thymic size [40], [15], [41] in the offspring and our study indicates that IL-7 in maternal milk contributes to this increased size. Both thymic output and peripheral proliferation contribute to the formation of the peripheral T cell pool [42], [43] and since proliferation expands only pre-existing peripheral T cells, greater thymic output would indicate a broader repertoire in the peripheral T cell pool. In addition to its effect on thymic size, provision of IL-7 from maternal sources would assist the proliferation of peripheral T cells. In adults the amount of available IL-7 in the periphery has been considered to regulate the size of the peripheral T cell pool, where its production by stromal cells in lymphoid organs appears to be uninfluenced by extrinsic stimuli [44]. The presence of maternally derived IL-7 will disturb the equilibrium between overall T cell numbers and the fixed rate of endogenous IL-7 production, introducing a bias towards the production of more T cells and supporting the survival of a larger peripheral T cell pool but only for a period limited both by the permeability of the gut and the availability of IL-7 within breast milk. High levels of IL-7 in milk in the period after birth when the integrity of the gut is incomplete could benefit the offspring by supporting the development and maintenance of a broad T cell repertoire at a time when the antigen load of the external environment increases substantially.
